# Preparation and Characterization of Acrylic Pressure-Sensitive Adhesives Crosslinked with UV Radiation-Influence of Monomer Composition on Adhesive Properties

**DOI:** 10.3390/ma15010246

**Published:** 2021-12-29

**Authors:** Karolina Mozelewska, Zbigniew Czech, Marcin Bartkowiak, Małgorzata Nowak, Paulina Bednarczyk, Piotr Niezgoda, Janina Kabatc, Agnieszka Skotnicka

**Affiliations:** 1 Department of Chemical Organic Technology and Polymeric Materials, Faculty of Chemical Technology and Engineering, West Pomeranian University of Technology in Szczecin, Piastow Ave. 42, 71-065 Szczecin, Poland; psa_czech@wp.pl (Z.C.); marcin.bartkowiak@zut.edu.pl (M.B.); gosia.nowak.zut@gmail.com (M.N.); bednarczyk.pb@gmail.com (P.B.); 2 Plant Protection Laboratory, Faculty of Environmental Management and Agriculture, West Pomeranian University of Technology in Szczecin, Piastów Ave. 42, 71-065 Szczecin, Poland; piotr.niezgoda@zut.edu.pl; 3 Department of Organic Chemistry, Faculty of Chemical Technology and Engineering, Bydgoszcz University of Science and Technology, Seminaryjna 3, 85-326 Bydgoszcz, Poland; janina.kabatc@wp.pl (J.K.); agnieszka.skotnicka@wp.pl (A.S.)

**Keywords:** pressure-sensitive adhesives, UV-C crosslinking, acrylate copolymers

## Abstract

In this study, syntheses of acrylate copolymers were performed based on the monomers butyl acrylate (BA), 2-ethylhexyl acrylate (2-EHA), and acrylic acid (AA) and the second-type unsaturated photoinitiator 4-acryloyloxybenzophenone (ABP). The structure of the obtained copolymers was confirmed via FT-IR spectroscopic analysis, and the viscosity and the content of non-volatile substances were determined. The adhesive films were then coated and cross-linked using ultraviolet radiation in the UV-C range at various doses (5–50 mJ/cm^2^). Due to the dependence of the self-adhesive properties of the adhesive layer on the basis weight, various basis weights of the layer in the range of 30–120 g/m^2^ were tested. Finally, the self-adhesive properties were assessed: tack, peel adhesion, shear strength (cohesion) at 20 °C and 70 °C, as well as the SAFT test and shrinkage. The aim of the study was to determine the effect of the type of monomer used, the dose of ultraviolet radiation, and the basis weight on the self-adhesive and usable properties of the obtained self-adhesive tapes.

## 1. Introduction

Acrylate-based pressure-sensitive adhesives are an interesting group of adhesives, among the most commonly used in industry. The documented history of adhesives goes back as far as 3000 BC, and the modern history of pressure-sensitive adhesives began in 1845, when a patent was granted for a self-adhesive mixture containing natural rubber and adhesive resins [1,2].

According to the modern definition, an adhesive is a substance capable of permanently bonding the surfaces of solids, which, after the evaporation of the solvent (solvent adhesives), water (dispersion adhesives), or cooling (hot-melt adhesives), form a self-adhesive layer of organic polymer on the substrate [3,4]. Among the pressure-sensitive adhesives used in the form of solvent-based, solvent-free, or water dispersions, polyacrylate adhesives have gained special importance due to their excellent properties. In recent years, a steady trend has been observed in the production and use of polyacrylate adhesives cross-linked by electron beam or UV rays [5,6,7].

Not every type of polymer can be used in the production of pressure-sensitive adhesives. The main criterion for determining whether a given type of polymer can be used as a macromolecular substance with self-adhesive properties is its glass transition temperature [8]. The most typical representatives of macromolecular compounds used in the production of self-adhesive materials, in addition to the previously discussed acrylate copolymers, are natural rubber, synthetic rubbers, copolymers of ethylene and acrylic acid, polyvinyl ethers, polyurethanes, polysiloxanes, and polyacrylates [9,10,11].

Due to the low production costs and the readily available technology, there is a particular interest in ultraviolet-ray cross-linked polyacrylate adhesives. Pressure-sensitive adhesives based on polyacrylates are characterized by very good tack; high cohesion; adhesion; low shrinkage; and good resistance to solvents, higher temperatures, and external conditions [12].

The functional properties of pressure-sensitive adhesives, and thus their use, largely depend on the type of adhesive used for their production. They can immediately adhere to various surfaces under the influence of a small pressure force and during a short contact period (a few seconds). Obtaining a durable and functional connection is made possible by meeting two basic conditions: achieving adequate strength in the adhesive joint (cohesion) and proper bonding of the adhesive joint with the surface of the joined material (adhesion). An important property of pressure-sensitive adhesives is also the tack, which allows immediate adhesion to the surface of the substrate under the influence of slight pressure, acting for a very short time [13,14,15].

The most important mechanical properties of pressure-sensitive adhesives depend on the viscoelastic properties of a given system (they should be between those of viscous liquids and ideal elastic bodies). The nature of the adhesive is determined by the balance between adhesion and cohesion determined for a given adhesive joint [16,17].

Special industry standards are used to determine the self-adhesive and functional properties of the adhesives and tapes obtained. The organization dealing with the definition of test methods for self-adhesive materials is FINAT (an international organization associating producers related to the production and use of self-adhesive labels)- a abbreviation of the French title: Féderation Internationale des fabricants et transformateurs d’Adhésifs et Thermocollants sur papiers et autres supports. As many as 31 research methods are recommended by FINAT, and their number is constantly increasing every year because of the progress in the self-adhesive materials industry. The research methods of this organization are defined by the letters FTM (Finat test method) and the numbers 1–30. Typically, customers of self-adhesive materials receive test data according to FTM 1–4, FTM 8–9, and FTM 12–13. Other studies are often included in the so-called industry research and generally, they are widely available only to a few clients [18,19,20].

In addition to FINAT, the AFERA standards (Association des Fabricants Européens de Rubans Auto-Adhésifs) are also used in the testing of self-adhesive products. These standards describe the methodology for performing basic tests, e.g., cohesion: AFERA 4012, tack: AFERA 4015 and peel adhesion: AFERA 4001 [21,22].

There are many publications on acrylate pressure-sensitive adhesives. Comparing the adhesive films obtained during this study, they are characterized by energy savings and the low volatility of organic compounds. The solvent used in the reaction is purified and reused. This is believed to be one of the most effective ways to rapidly convert the solventless adhesive film into a solid polymer at ambient temperature. Under intense lighting, the cross-linking of the films is very intense, and sometimes in a fraction of a second they form a dense three-dimensional polymer network that exhibits excellent resistance to chemicals, heat, and organic solvents [23,24].

The main aim of the study was to determine the influence of the type of monomer and its amount on the self-adhesive properties of adhesive film Syntheses with butyl acrylate, 2-ethylhexyl acrylate, acrylic acid, and a type II unsaturated photoinitiator (ABP) were performed. The next stage of the research was to determine the most effective dose of cross-linking with UV-C radiation. Moreover, the influence of the thickness of the adhesive layer on the self-adhesive properties of the obtained products was also investigated.

## 2. Materials and Methods

### 2.1. Materials

In order to obtain photoreactive pressure-sensitive adhesives, a free radical polymerization reaction was performed. Commonly available technically pure reagents were used. The following ingredients were used in the tests: 2-ethylhexyl acrylate (2-EHA) (POLY-CHEM GmbH, Bitterfeld-Wolfen, Germany), butyl acrylate (BA), acrylic acid (AA) and ethyl acetate were purchased from BASF (Ludwigshafen, Germany) and AIBN (2,2′-azo-diisobutyonitrile) Vazo 65 from Union Carbide (Houston, TX, USA), as well as 4-acryloyloxy benzophenone (ABP) (Chemitec, Scandiccy, Italy). Acrylate monomers of technical purity were used, whereas the photoinitiator and initiator had a purity of 99.5%.

The functions and the chemical formulas of the compounds used are presented in Table 1. In the presented tests, 4-acryloyloxy benzophenone (ABP) was used as a photoinitiator. On the other hand, the role of the radical initiator was served by AIBN. Table 1 shows the composition of the synthesized photoreactive adhesive.

### 2.2. Synthesis of Acrylic Pressure-Sensitive Adhesives

The pressure-sensitive adhesive was synthesized in ethyl acetate (50 wt.% polymer content) with 95 wt.% butyl acrylate/2-ethylhexyl acrylate and 5 wt.% acrylic acid. Both butyl acrylate and 2-ethylhexyl acrylate were used in the synthesis of P3.

**Figure 1:**
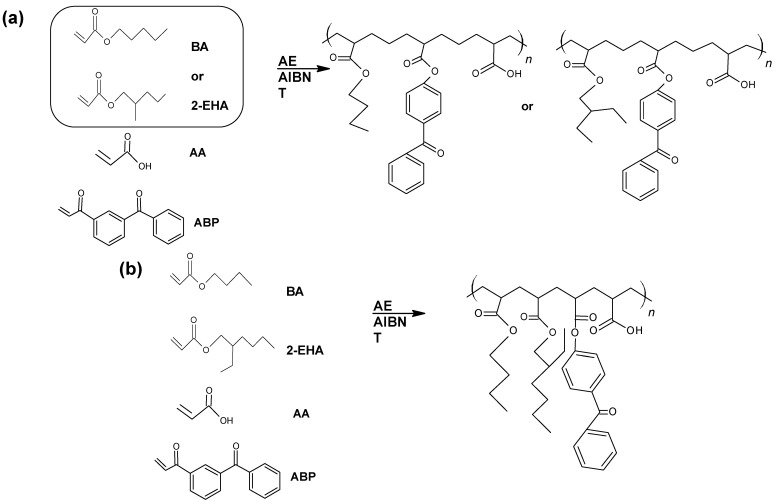
Schematic presentation of the performed syntheses and theoretical structures of the synthesized copolymers ((**a**) P1 and P2, (**b**) P3). (AE-ethyl acetate).

Free-radical polymerization was carried out in a glass reactor with a capacity of 250 cm^3^, immersed in an oil bath, equipped with a mechanical stirrer, a reflux condenser, and a dosing system (100 mL addition funnel). The process was carried out at the boiling point of the solvent, at about 77 °C. The AIBN initiator was dissolved in the monomer mixture and added dropwise to the reactor over a strictly defined time (dosing time). The actual polymerization was then carried out. During the entire process, the stirrer’s rotation was 170 revolutions/min. The reaction conditions and the basic properties of the acrylic copolymers are shown in Table 2. The theoretical structures of the obtained acrylic copolymers are shown in Figure 1.

### 2.3. Preparation of Self-Adhesive Tapes

**Figure 2:**
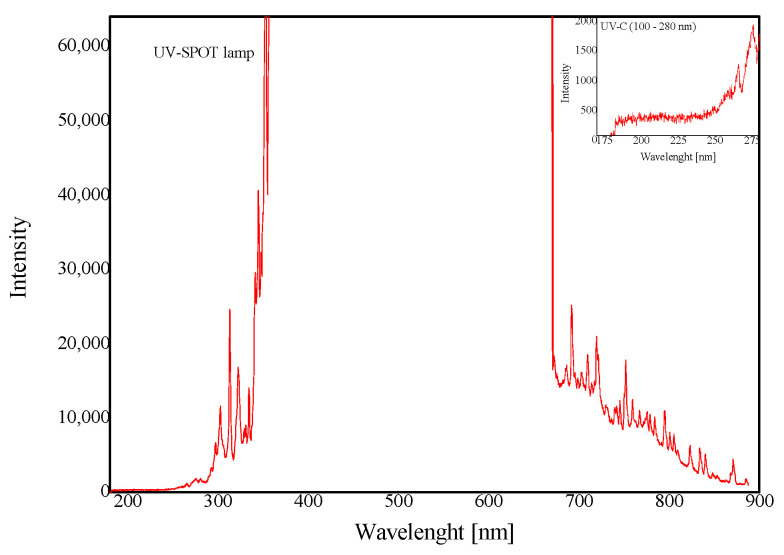
The wavelength range of radiation emitted by the UV lamp used.

The obtained acrylate adhesive was coated on a polyester film with a thickness of 50 g/m^2^ using a semi-automatic PSAT coater. In order to determine the influence of the basis weight of the adhesive layer on the self-adhesive properties of the obtained tapes, the obtained adhesive was coated with the following basis weights: 30, 60, 90, and 120 g/m^2^. Then, it was dried in a laboratory oven for 10 min at 110 °C. The next step was to cross-link the adhesive film with the use of UV-C radiation with a different radiation dose in the range of 5–50 mJ/cm^2^. For cross-linking, a UV lamp—UVASPOT by Honle (Munich, Germany) with an intensity of approx. 400 mW /cm^2^—was used. The absorption range of the lamp is shown in Figure 2. The conditions of cross-linking with the use of UV-C radiation and the evaporation of the solvent are presented in Table 3.

### 2.4. Methods

The properties of pressure-sensitive adhesives depend to a large extent on their viscoelastic properties (intermediate between viscous liquids and ideal elastic bodies) for a given system. In the presented work, the following properties were tested: adhesion (peel adhesion), tack (initial adhesion), cohesion (internal shear strength of the crosslinked adhesive layer), and thermal resistance. In the case of an adhesive suitable for use in industry, it is important to maintain a balance between cohesion and adhesion [25,26].

### 2.5. Characterization of the Pressure-Sensitive Adhesives

The dynamic viscosity was determined using a DV-II Pro Extra viscometer (Brookfield, New York, NY, USA). The solids content was determined using a moisture analyzer (Radwag MAX 60/NP, Radom, Poland). After evaporating the solvent, the samples were heated in aluminum crucibles at the temperature of 140 °C for 40 min. The coat weight of the obtained adhesive film was determined using a round punch with an area of 10 cm^2^ (Karl Schröder KG, Weinheim, Germany). Infrared spectra were obtained using a Nicolet iS5 instrument (Thermo Fisher, Waltham, MA, USA). The scanning range was 400–4000 cm^−1^, whereas the resolution of the device was 4 cm^−1^.

### 2.6. Characterization of the Adhesive Tape

#### 2.6.1. Peel Adhesion Testing

Peel adhesion measurement was performed on a Zwick/Roell Z010 testing machine (Ulm, Germany) at room temperature. Cross-linked, adhesive tapes measuring 2.5 × 12.7 cm were stuck to a standard steel plate. After 20 min, the plate was placed in the lower holder of the testing machine, whereas the other part of the tape was placed horizontally in the upper holder of the testing machine. The test involved removing the adhesive tape at an angle of 180° with a jaw speed of 300 mm/min according to the international standard AFERA 4001. During the measurement, the force that must be used to peel the adhesive film from the steel plate was measured [27,28].

#### 2.6.2. Cohesion Test

The cohesion test of the obtained adhesive tapes was tested at room temperature and elevated temperature (70 °C), according to the international FINAT FTM 8 method. The 2.5 × 2.5 cm adhesive film was applied to the steel plate and after about 10 min the other end of the strip was loaded with a weight with a mass of 1 kg. According to the standard, cohesion is the time after which the adhesive film will separate from the steel plate, under a load of 1 kg [29,30].

#### 2.6.3. SAFT test

In the SAFT test, the adhesive tape sample was prepared in the same way as above. The difference, however, is that the temperature during the test was not constant, increasing from 20 °C to 220 °C with a temperature increase of 1.5 °C/min. The measurement carried out in this way was intended to determine the time and temperature at which the test sample would fall off the plate [27].

#### 2.6.4. Tack

The tack of the obtained self-adhesive tapes was tested following the FTM9 standard using a Zwick/Roell Z010 testing machine. These tests were carried out in the following way: a tape strip (dimensions: 2.5 × 17.5 cm) was placed in the jaws of a testing machine in the form of a loop. Then the upper jaw of the machine was lowered, causing brief contact between the adhesive tape and the plate. During the measurements, the force required to detach the adhesive tape from the metal plate was measured [31].

#### 2.6.5. Shrinkage

**Figure 3:**
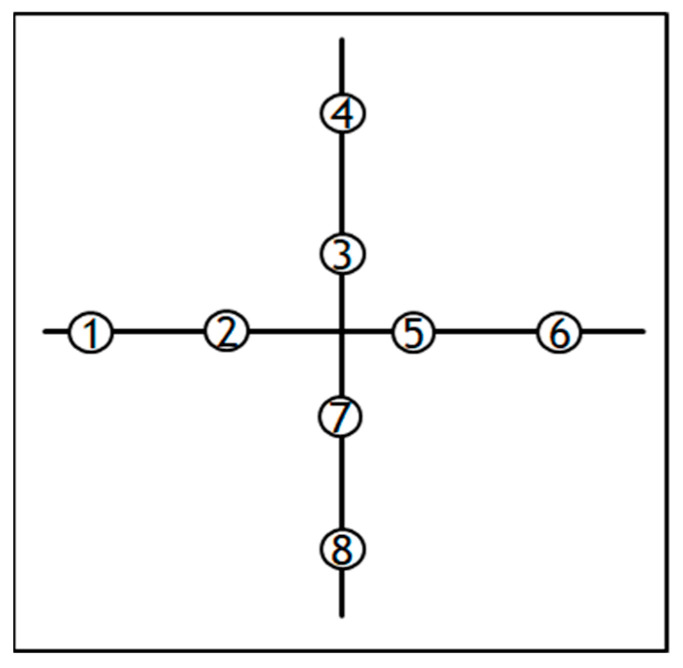
Conducting a shrinkage test using the cross method.

The shrinkage of pressure-sensitive adhesives is defined by observing the reduction in its size to half of its original size. It is closely related to the cross-linking process (its type and cross-linking compound). This is an important property of pressure-sensitive adhesives, especially in applications where shrinkage can affect the surface of the pressure-sensitive adhesive and cause it to deform. For pressure-sensitive adhesives, shrinkage means a percentage or millimeter change in the dimension of the film coated with the adhesive. A PVC (poly vinyl chloride) or PET (poly(ethylene terephthalate). film is used, which is stuck to a metal plate, and two incisions are made (Figure 3) and the adhesive film is placed at a temperature of 70 °C. Then, after a certain period of time, changes in the size of the film are checked at eight different places. The result of the test is the arithmetic mean of all points. The shrinkage value above 0.5 mm or 0.5% exceeds the allowable value in the technology of pressure-sensitive adhesives [32].

## 3. Results

**Figure 4:**
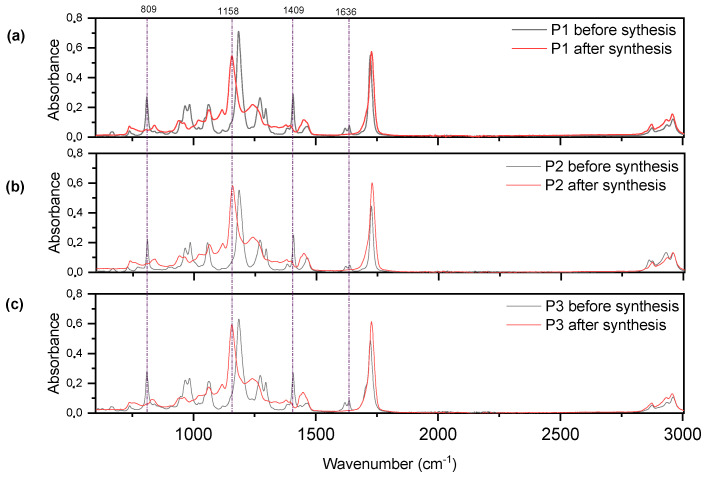
FTIR spectra of the reaction mixture before the copolymerization and the obtained product after the reaction for (**a**) P1, (**b**) P2, and (**c**) P3.

Using FTIR infrared spectroscopy, we determined how the copolymerization reaction takes place. Figure 4 shows the FTIR spectra of the reaction mixture before the reaction and the FTIR spectra after the reaction (of the obtained pressure-sensitive adhesive). The disappearance of the peaks was observed at the wavelengths 809 cm^−1^, 1409 cm^−1^, and 1636 cm^−1^ (corresponding C = C bonds), confirming that acrylate groups are involved in the reaction and that the copolymerization reaction is taking place. After synthesis, the appearance of the 1158 cm^−1^ peak was observed, which may be due to the appearance of ester bonds.

In the presented work, the influence of various monomers on self-adhesive properties was investigated, taking into account:

- The thickness of the adhesive tape: 30, 60, 90 and 120 g/m^2^;
- The UV-C radiation dose: 5, 10, 20, 30 and 50 mJ/cm^2^.

**Figure 5:**
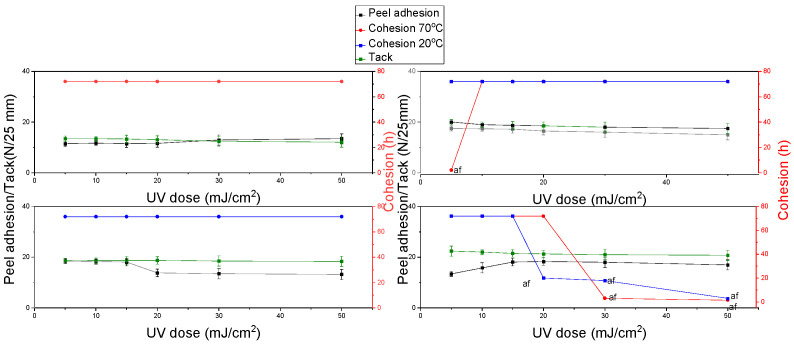
Results of cohesion at 20 °C and 70 °C, peel adhesion, and tack for self-adhesive tapes obtained from acrylate copolymers from P1 synthesis.

Figure 5 shows the results of cohesion at 20 °C and 70 °C, as well as adhesion and tack with respect to different basis weights and cross-linking doses. In the case of cohesion, regardless of the temperature, the maximum value was observed above 72 h. According to literature reports, the cohesion value decreases with increasing coating weight [24,33]. In the case of the P1 adhesive, the samples showed the maximum value of cohesion at 72 h (for 30, 60 and 90 g/m^2^), whereas the lower value was observed for the basis weight of 120 g/m^2^.This may be due to too little ultraviolet radiation penetrating through thicker layers, which results in partial cross-linking of the adhesive. Tack and cohesion show high values, which increase with increasing the basis weight of the adhesive layer and decrease with increasing the dose of UV-C radiation.

**Figure 6:**
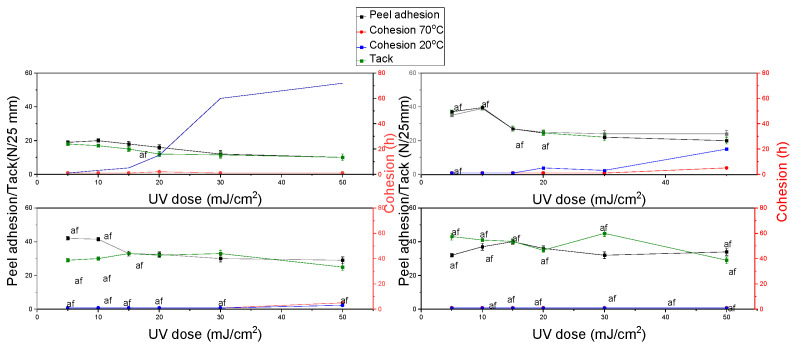
Results of cohesion at 20 °C and 70 °C, peel adhesion, and tack for self-adhesive tapes obtained from acrylate copolymers from P2 synthesis.

The results for self-adhesive tapes obtained from acrylate copolymers from P2 synthesis are shown in Figure 6. Cohesion at the temperatures of 20 °C and 70 °C showed low values, which are unacceptable in the technology of pressure-sensitive adhesives. Only for the basis weight of 30 g/m^2^ and the radiation dose of 50 mJ/cm^2^, the higher cohesion at a temperature of 20 °C can be observed. Tack and peel adhesion showed high values, but a cohesive failure was observed during the test. This may be due to the lack of cross-linking of tapes made predominantly of an acrylate copolymer based on 2-ethylhexyl acrylate.

**Figure 7:**
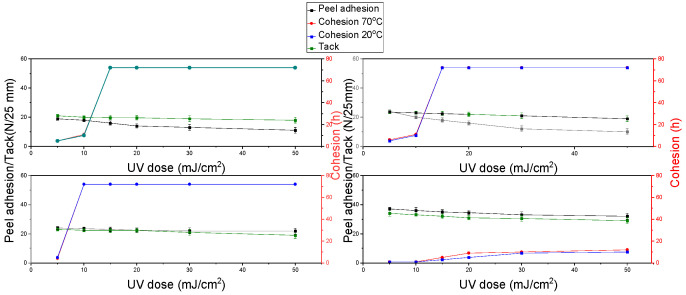
Results of cohesion at 20 °C and 70 °C, peel adhesion, and tack for self-adhesive tapes obtained from acrylate copolymers from P3 synthesis.

Figure 7 shows the results for the tapes obtained from the copolymer from the P3 synthesis. These tapes showed high tack and peel adhesion results, even above 30 N/25 mm. The maximum value of cohesion, above 72 h, was also observed for tapes cross-linked with a higher radiation dose. In the case of pressure-sensitive adhesives, an important aspect is to maintain a balance between adhesion to the substrate and the internal strength of the adhesive layer. Therefore, the copolymer synthesized in the P3 polymerization had the best properties (peel adhesion, cohesion, and tack).

**Figure 8:**
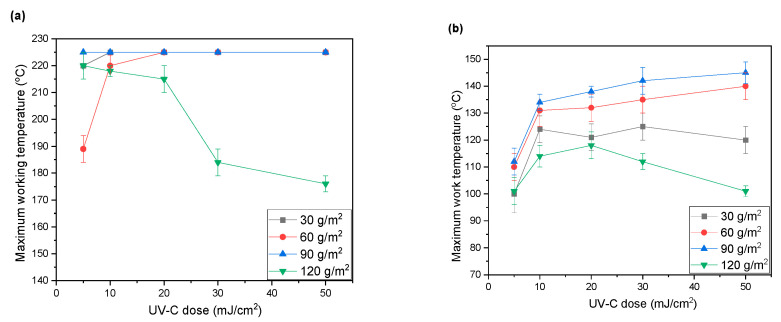

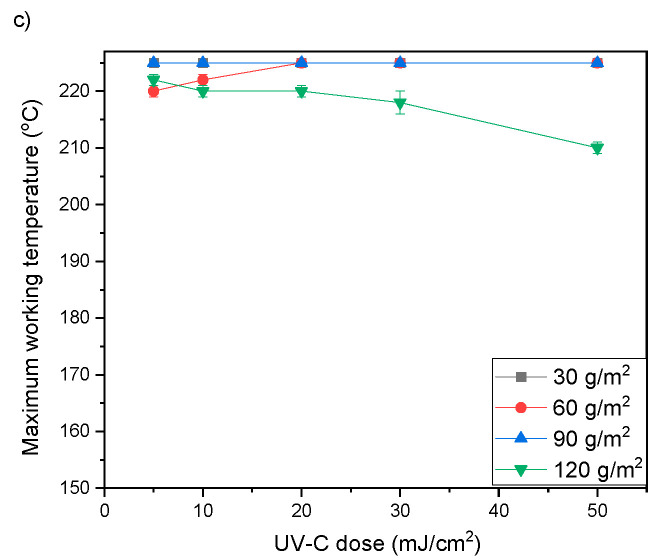
Maximum work temperature of tapes for self-adhesive tapes obtained from acrylate copolymers (**a**) from P1 synthesis, (**b**) from P2 synthesis, and (**c**) from P3 synthesis.

In order to determine the maximum operating temperature, the obtained self-adhesive tapes were subjected to the SAFT test. The results are shown in Figure 8. All obtained products were characterized by high thermal resistance, but the highest results were obtained for the acrylate copolymer obtained in the reaction of P3 with butyl acrylate and 2-ethylhexyl acrylate (Figure 8c). Furthermore, tapes made of copolymer with butyl acrylate showed high thermal resistance (Figure 8a). The lowest results were obtained for the acrylate copolymer from reaction P2 (Figure 8b). For all the copolymers obtained, the maximum working temperature was the lowest for the basis weight of 120 g/m^2^. For the remaining basis weights, the values were high. In the case of an increase in the radiation dose, the maximum operating temperature increased (for the P2 copolymer), whereas in other cases it was difficult to find a relationship. This may have been caused by the use of an insufficient radiation dose, and thus an overly short exposure time, and an overly thick adhesive layer, which would make it difficult for radiation to penetrate the entire adhesive layer.

Table 4 shows the results of testing the shrinkage of the manufactured tapes. Table 4a shows the results for the copolymer produced in the P1 reaction, in which the main monomer was butyl acrylate. They are characterized by the highest values, compared to tapes made of copolymer based on 2-ethylhexyl acrylate (Table 4b). The lowest shrinkage was recorded for tapes made of the P3 copolymer, in which the monomers were both 2-ethylhexyl acrylate and butyl acrylate (Table 4c). The shrinkage values increase with time, but stabilize after a certain cut-off time, which is shorter for cross-linked tapes at lower dosages. As the radiation dose increases, the contraction decreases. This phenomenon may result from better cross-linking of the adhesive layer at higher doses of UV-C radiation.

## 4. Conclusions

In the presented work, acrylate copolymers capable of cross-linking with the use of UV-C radiation were obtained. The incorporation of an unsaturated photoinitiator monomer into the copolymer chain results in high self-adhesive parameters. The exceptionally high efficiency of ABP as a copolymerizing photoinitiator is attributed to, inter alia, a short ABP molecule and the short distance between both cross-linked polymer chains after UV irradiation.

The acrylate copolymers obtained in the P3 reaction based on acrylate acid, 2-ethylhexyl acrylate, butyl acrylate, and ABP showed the best self-adhesive properties and the lowest shrinkage. The tested UV-crosslinked photoreactive acrylic pressure-sensitive adhesives can be used to produce high-performance self-adhesive materials such as mounting tapes, labels, masking tapes, or signs and marking foils.

## Figures and Tables

**Table 1:** Characteristics of monomers and other reagents used for syntheses.

Monomer	Structure	Function
Acrylic acid (BASF)	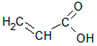	Crosslinking monomer
Butyl acrylate (BASF)	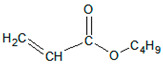	It improves the self-adhesive properties: cohesion
2-ethylhexyl acrylate (POLY-CHEM)	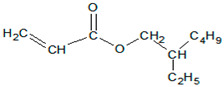	It improves self-adhesive properties: peel adhesion
AIBN Vazo 65 (Union Carbide)	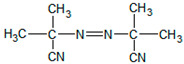	Thermal radical initiator
ABP 4-acryloyloxy benzophenone (Chemitec)	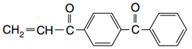	Photoinitiator
Ethyl acetate (BASF)	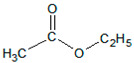	Polymerization medium

**Table 2:** Composition of synthesized pressure-sensitive adhesives and reaction conditions. Monomers: acrylic acid (AA), 2-ethylhexyl acrylate (2-EHA), butyl acrylate (BA).

Name	Monomer Formulation	Wt.% Ratio	ABP (wt.%)	Initiator (wt.%)	Dosage Time (h)	Reaction Time (h)	η (Pa·s)	^1^ SWC (wt.%)
P1	BA:AA	95:5	0.5	AIBN 0.1	1.5	5	2.6	95.1
P2	2-EHA:AA	95:5					25.4	98.7
P3	2-EHA:BA:AA	47.5:47.5:5					14.5	97.1

^1^ SWC, solid weight content.

**Table 3:** The conditions of preparation of adhesive films for P1 synthesis. The films from the remaining syntheses were made analogously.

Name	ES ^1^: Time (min)/Temperature (°C)	Coat Weight ^2^ (g/m^2^)	UV-C Dose (mJ/cm^2^)	UV-Crosslinking Time (s)
P1-30-5	10/110	30	5	1
P1-30-10			10	2
P1-30-20			20	4
P1-30-30			30	6
P1-30-50			50	10
P1-60-5	10/110	60	5	1
P1-60-10			10	2
P1-60-20			20	4
P1-60-30			30	6
P1-60-50			50	10
P1-90-5	10/110	90	5	1
P1-90-10			10	2
P1-90-20			20	4
P1-90-30			30	6
P1-90-50			50	10
P1-120-5	10/110	120	5	1
P1-120-10			10	2
P1-120-20			20	4
P1-120-30			30	6
P1-120-50			50	10

^1^ ES, evaporation of solvent; ^2^ coat weight of adhesive film.

**Table 4:** Shrinkage of obtained copolymers depending on the dose of UV-C crosslinking (**a**) P1, (**b**) P2, and (**c**) P3. The measurement error was within the range of the statistical error.

**(a)**												
**Shrinkage (%)**												
**UV-Dose** **(mJ/cm^2^)**	**10 min**	**30 min**	**1 h**	**3 h**	**8 h**	**24 h**	**2 Days**	**3 Days**	**4 Days**	**5 Days**	**6 Days**	**7 Days**
5	0.114	0.122	0.224	0.289	0.308	0.389	0.401	0.523	0.601	0.601	0.601	0.601
10	0.178	0.221	0.255	0.339	0.354	0.404	0.504	0.546	0.562	0.588	0.672	0.672
20	0.157	0.116	0.201	0.226	0.301	0.339	0.368	0.446	0.454	0.468	0.508	0.508
30	0.147	0.192	0.226	0.255	0.263	0.318	0.321	0.384	0.341	0.362	0.467	0.48
50	0.15	0.163	0.184	0.202	0.213	0.249	0.27	0.289	0.291	0.307	0.341	0.355
**(b)**												
**Shrinkage (%)**												
**UV-Dose** **(mJ/cm^2^)**	**10 min**	**30 min**	**1 h**	**3 h**	**8 h**	**24 h**	**2 Days**	**3 Days**	**4 Days**	**5 Days**	**6 Days**	**7 Days**
5	0.241	0.256	0.274	0.321	0.399	0.409	0.452	0.523	0.599	0.599	0.599	0.599
10	0.171	0.197	0.389	0.417	0.48	0.501	0.53	0.546	0.567	0.651	0.688	0.759
20	0.155	0.239	0.378	0.397	0.455	0.459	0.483	0.489	0.491	0.517	0.538	0.538
30	0.096	0.121	0.126	0.163	0.191	0.2	0.218	0.279	0.339	0.362	0.425	0.492
50	0.087	0.097	0.097	0.105	0.134	0.147	0.213	0.262	0.271	0.346	0.368	0.373
**(c)**												
**Shrinkage (%)**												
**UV-Dose** **(mJ/cm^2^)**	**10 min**	**30 min**	**1 h**	**3 h**	**8 h**	**24 h**	**2 Days**	**3 Days**	**4 Days**	**5 Days**	**6 Days**	**7 Days**
5	0.115	0.124	0.224	0.250	0.301	0.314	0.391	0.408	0.436	0.448	0.448	0.448
10	0.114	0.114	0.214	0.256	0.321	0.336	0.381	0.389	0.439	0.446	0.458	0.458
20	0.102	0.107	0.202	0.241	0.314	0.324	0.372	0.379	0.440	0.447	0.476	0.479
30	0.100	0.106	0.145	0.234	0.302	0.314	0.351	0.368	0.374	0.421	0.435	0.452
50	0.097	0.101	0.174	0.181	0.201	0.221	0.241	0.253	0.268	0.297	0.322	0.335

## Data Availability

Not applicable.
